# Active Region Mode Control for High-Power, Low-Linewidth Broadened Semiconductor Optical Amplifiers for Light Detection and Ranging

**DOI:** 10.3390/s24186083

**Published:** 2024-09-20

**Authors:** Hui Tang, Meng Zhang, Lei Liang, Tianyi Zhang, Li Qin, Yue Song, Yuxin Lei, Peng Jia, Yubing Wang, Cheng Qiu, Chuantao Zheng, Xin Li, Yongyi Chen, Dan Li, Yongqiang Ning, Lijun Wang

**Affiliations:** 1 Key Laboratory of Luminescence Science and Technology, Chinese Academy of Sciences & State Key Laboratory of Luminescence and Applications, Changchun Institute of Optics, Fine Mechanics and Physics, Chinese Academy of Sciences, Changchun 130033, China; htang2024@sinano.ac.cn (H.T.); zhangmeng223@mails.ucas.ac.cn (M.Z.); qinl@ciomp.ac.cn (L.Q.); songyue@ciomp.ac.cn (Y.S.); leiyuxin@ciomp.ac.cn (Y.L.); jiap@ciomp.ac.cn (P.J.); wangyubing@ciomp.ac.cn (Y.W.); qiucheng@ciomp.ac.cn (C.Q.); ningyq@ciomp.ac.cn (Y.N.); wanglj@ciomp.ac.cn (L.W.); 2 Suzhou Institute of Nano-Tech and Nano-Bionics (SINANO), Chinese Academy of Sciences, Suzhou 215123, China; 3 Peng Cheng Laboratory, No. 2, Xingke 1st Street, Shenzhen 518000, China; chenyy@ciomp.ac.cn; 4 Jilin Changguang Jixin Technology Co., Ltd., No. 206, Software Road, HTDZ, Changchun 130022, China; 18943085572@163.com; 5 State Key Laboratory on Integrated Optoelectronics, College of Electronic Science and Engineering, Jilin University, Changchun 130012, China; zhengchuantao@jlu.edu.cn; 6 Jlight Semiconductor Technology Co., Ltd., No. 1588, Changde Road, ETDZ, Changchun 130102, China; 7 National Key Laboratory of Advanced Vehicle Integration and Control, China FAW Corporation Limited, No. 1, Xinhongqi Street, Changchun 130000, China; lidan@faw.com.cn

**Keywords:** semiconductor optical amplifier, active region mode control, high power, low-linewidth broadening, low polarization-dependent gain

## Abstract

This paper introduces a semiconductor optical amplifier (SOA) with high power and narrow linewidth broadening achieved through active region mode control. By integrating mode control with broad-spectrum epitaxial material design, the device achieves high gain, high power, and wide band output. At a wavelength of 1550 nm and an ambient temperature of 20 °C, the output power reaches 757 mW when the input power is 25 mW, and the gain is 21.92 dB when the input power is 4 mW. The 3 dB gain bandwidth is 88 nm, and the linewidth expansion of the input laser after amplification through the SOA is only 1.031 times. The device strikes a balance between high gain and high power, offering a new amplifier option for long-range light detection and ranging (LiDAR).

## 1. Introduction

Optical amplifiers, as a type of laser device, are primarily used to amplify optical signals, enhancing the detection range and accuracy of optical sensors. In coherent LiDAR applications, high-power optical amplifiers operating in the 1550 nm wavelength band are commonly employed due to their relatively high eye-safe power thresholds, enabling long-distance coherent detection. For vehicle-mounted LiDAR systems, which are central to achieving L4 and L5 autonomous driving technology, high-power optical amplifiers are necessary to support forward detection distances of up to 200 m. In this context, small, easy-to-integrate, and cost-effective SOAs present a promising new option for cost-sensitive vehicle-mounted LiDAR systems [1,2,3,4].

However, current commercially available SOAs have a low output power, typically only in the range of hundreds of milliwatts [5,6,7]. It is difficult for high-power devices to achieve a small-signal gain exceeding 20 dB, and similarly, high-gain devices struggle to surpass 500 mW in power. Based on this, the present paper proposes a high-power, low-linewidth broadening SOA operating at 1550 nm, utilizing active region mode control. Through a dual-stage cascaded mode control employing narrow stripes and tapered waveguides, active region optical field confinement factor modulation is achieved, enabling a high-gain, high-power, and broad bandwidth output.

## 2. Device Structure Design and Preparation Process

### 2.1. The Comprehensive Design of the Device’s Structure

**Figure 1:**
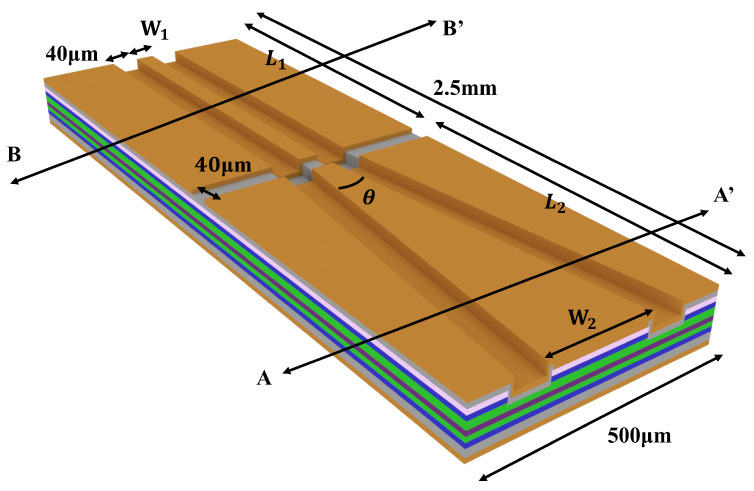
A schematic of the structure of the SOA device.

The proposed device structure is designed to create a high-power SOA device with controllable optical field modes (see Figure 1). The device consists of two main parts: the first part is the high-gain region with a high confinement factor of the active region optical field, characterized by a length of L_1_ = 1.0 mm and a ridge width of W_1_ = 4.0 μm, providing a high gain for the fundamental transverse mode; the second part is the high-power region, separated from the first part by electrodes isolated by grooves with a width of 40 μm. The waveguide length is denoted as L_2_ = 1.5 mm with the width gradually expanding from W_1_ to W_2_ = 250 μm at an angle of θ = 10.4° for the tapered structure. The maximum output power is enhanced through lateral mode expansion.

**Figure 2:**
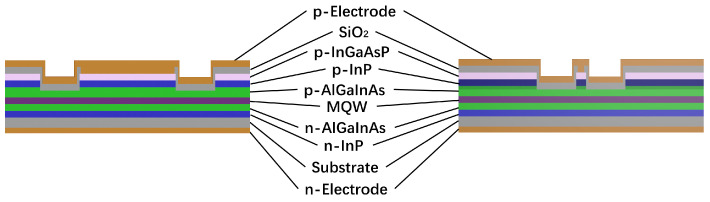
A schematic of the epitaxial structure of the device along the cross-sections AA’ (**left**) and BB’ (**right**).

The active region is used for epitaxial material growth on an InP substrate using a metal-organic chemical vapor deposition (MOCVD) system. The active region is a compressively strained quantum well structure consisting of five 6 nm thick Al_0.0696_GaInAs quantum wells and six 10 nm thick Al_0.2244_GaInAs barriers (see Figure 2). The active region is positioned between three n-type graded AlGaInAs lower cladding layers and two p-type graded AlGaInAs upper cladding layers. The detailed structure is shown in Table 1. The preparation of such complex structures depends on the precise tuning of the MOCVD. The preparation of this complex epitaxial structure depends on the precise control of the thickness, composition, and doping concentration of the epitaxial materials achieved by using a MOCVD system. For material thickness control, the error is usually required to be less than 5%, and the smaller the percentage, the better. We achieved this goal by using the in situ monitoring method of epitaxial reflectance anisotropy spectroscopy (Epi-RAS); this can also be achieved by continuously and accurately calibrating the material growth rate. In the actual growth process, Epi-RAS monitoring requires real-time artificial intervention operations such as pauses and jumps during epitaxial growth. Therefore, we used scanning electron microscopy (SEM) and superlattice X-ray diffraction (XRD) for the joint analysis and calibration of the material growth rate, thereby achieving high-precision control of the material growth thickness. In terms of material component control, real-time calibration experiments on the components are required by controlling the growth conditions, such as growth temperature, reaction chamber pressure, V/III ratio, rotation speed, etc., and then performing X-ray diffraction curve testing to analyze the parameters, such as a lattice mismatch. After multiple iterations of growth and testing, the target material with an extremely high lattice matching degree is finally obtained. For the doping concentration control, with help from the electrical characteristics as a function of the voltage (E-CV) curve, precise control of material doping concentration growth can be easily achieved. This quantum well structure exhibits a low optical field confinement factor and allows the mode field to expand toward the lower cladding layers, thus enhancing the saturated output power.

### 2.2. The Design of the Epitaxial Structure

To achieve a high-power amplifier, it is essential to balance the saturated output power and small-signal gain. The small-signal gain G_0_ represents the gain when the SOA is operating in the linear region and can be expressed as follows [8]:

(1) $$G_{0}=\exp[(\Gamma g_{0}-\beta )L].$$

where g_0_ represents the material gain, β represents the internal loss, and L represents the device cavity length. The saturated output power P_sat_ represents the output power when the linear region gain of the SOA decreases to 1/N_de_, which can be expressed as follows [8]:

(2) $$P_{sat,N}=\frac{(N_{de}/10)\ln10}{\left(1-\frac{10^{N_{de}/10}}{G_{0}}\right)}P_{s}.$$

**Figure 3:**
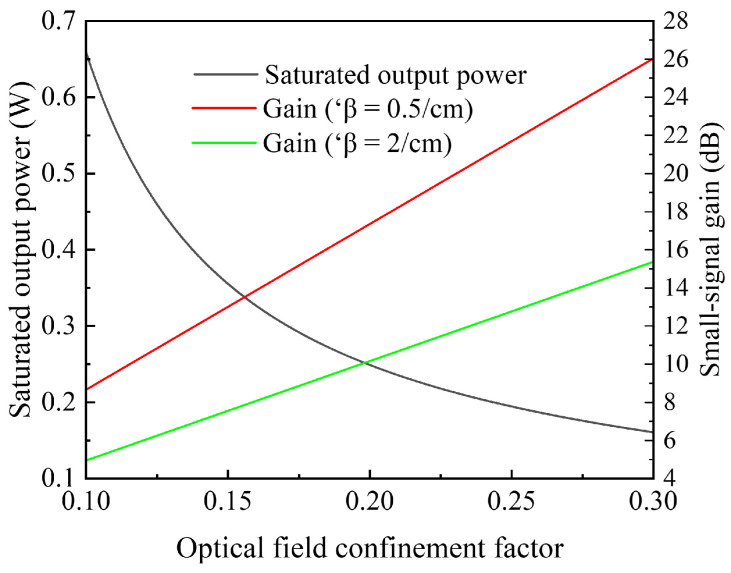
The simulation curves of the relationship between the saturated output power P_sat_ (3 dB), small-signal gain G_0_, and the optical field confinement factor Γ.

The constant N_de_ represents the decrease in gain in the linear region of the SOA in N_de_ dB. The most commonly used values for N_de_ are 3 and 10, indicating a 3 dB and 10 dB decrease in gain, respectively. P_s_ is the maximum output power that the epitaxial structure can withstand, which can be expressed as follows [8]:

(3) $$P_{s}=(\frac{wd}{\Gamma })(\frac{h\nu }{\alpha \tau }).$$

where Γ represents the optical field confinement factor, w represents the device waveguide width, d represents the whole thickness of the active region of the device, and α represents the differential gain of the material. Taking the ridge waveguide structure as an example, the simulation relationship curves among these factors are illustrated in Figure 3, where d = 90 nm and w = 6 μm. To ensure the accuracy of the simulation results, we assume that the differential gain α = 10^−16^ cm^2^ and carrier lifetime τ = 10^−9^ s do not vary with the carrier concentration. As shown in the figure, the P_sat_ (3 dB) decreases exponentially with an increase in Γ. To achieve a P_sat_ greater than 0.6 W, the Γ needs to be less than 0.1056. At this point, regardless of whether the internal loss β is 0.5 cm^−1^ or 2 cm^−1^, the small-signal gain of the device is less than 10 dB. The G_0_ increases linearly with the Γ. To achieve a small-signal gain greater than 20 dB, the Γ needs to be greater than 0.2306, or the device cavity length L needs to be further extended.

Therefore, it is challenging for a single-ridge waveguide device to achieve a watt-level saturated output power while maintaining a small-signal gain over 20 dB. Hence, the device in this study adopts a two-stage structure in its design. In the first stage, the ridge waveguide enhances gain in the fundamental transverse mode through a high optical confinement factor. In the second stage, the tapered waveguide expands the mode field and enhances the device’s saturated output power through a low optical confinement factor.

**Figure 4:**
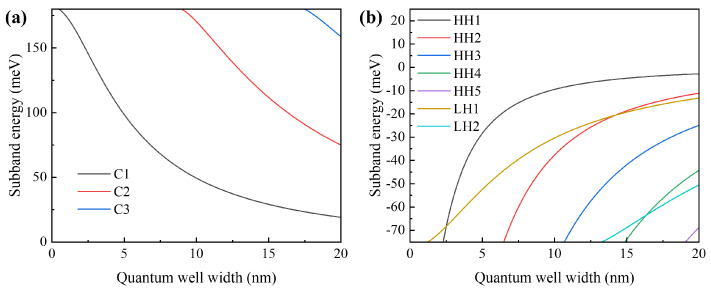
**The** simulation curves of electron subband energy and (**a**) heavy-hole subband energy versus (**b**) quantum well width L_w_.

The quantum well width *L_w_* stands out as one of the pivotal parameters in epitaxial design (see Figure 4). From the picture, it can be observed that the electron subband energy decreases with an increase in *L_w_*, while the hole subband energy increases with an increase in *L_w_*. This indicates that wider quantum wells can lead to smaller bandgap energy differences, thereby enhancing the material gain of SOAs, to a certain extent. However, larger *L_w_* can also limit the exciton effect, increase non-radiative recombination, and weaken the quantum size effect.

Therefore, considering these factors comprehensively, a quantum well width of 6 nm was adopted. At this width, the primary electron subband in the quantum well is the C1 subband, and the primary hole subbands are the heavy-hole subband HH1 and the light-hole subband LH1, with the energy of the heavy-hole subband HH2 being relatively low. Consequently, only the HH1 and LH1 subbands are primarily involved in photon coupling, facilitating a single-mode amplification of the waveguide. Furthermore, the increase in energy of the LH1 subband is beneficial for enhancing the mode gain of the TM mode, thereby reducing the polarization-dependent gain (PDG) of the device.

Ideally, the gain coefficient g of SOA materials, neglecting carrier losses, can be mathematically expressed as follows [9]:

(4) $$g(\hbar \nu )=\frac{{m_{r}}^{*}e^{2}}{n_{r}c\varepsilon_{0}{m_{0}}^{2}\nu \hbar^{2}L_{z}}{\int_{0}^{\infty }dE_{t}\;|\;\overset{\wedge }{e}*p_{cv}\;|\;}^{2}\frac{\gamma /\pi }{{\left[E_{h}^{e}+E_{t}-\hbar \nu \right]}^{2}+\gamma^{2}}\times \left[f_{c}\left(E_{t}\right)-f_{v}\left(E_{t}\right)\right].$$

(5) $$\left\{\begin{array}{c} f_{c}(E_{t})=\frac{1}{1+\exp\left[\left(E_{g}+E_{e}+\frac{{m_{r}}^{*}}{{m_{e}}^{*}}E_{t}-F_{c})/(k_{B}T\right)\right]}.\\ f_{v}(E_{t})=\frac{1}{1+\exp\left[\left(E_{h}-\frac{{m_{r}}^{*}}{{m_{h}}^{*}}E_{t}-F_{v})/(k_{B}T\right)\right]}. \end{array}\right.$$

(6) $$\frac{1}{{m_{r}}^{*}}=\frac{1}{{m_{e}}^{*}}+\frac{1}{{m_{h}}^{*}}.$$

where *ℏ* represents the reduced Planck’s constant, *ν* is the input signal optical frequency, *e* represents the electron charge, *n_r_* represents the refractive index, *c* represents the speed of light in a vacuum, *ε*_0_ represents the vacuum capacitance, *m*_0_ represents the electron mass, *L_z_* represents the thickness of the quantum well, ${\left|\hat{e}\;*\;p_{cv}\right|}^{2}$ represents the momentum matrix element, *γ* represents the refractive index diffusion factor, *E_t_* represents the energy level complex center, $E_{h}^{e}$ represents the band-side jump energy, *E_g_* represents the bandgap energy, m_e_^*^ and m_h_^*^ represent the approximate masses of the conduction-band electrons and valence-band holes, *E_e_* and *E_h_* represent the energy levels of the electrons and holes, *F_c_* and *F_v_* represent the quasi-Fermi energy levels of the electrons and holes, *k_B_* represents the Boltzmann constant, and *T* represents the reference temperature. It is worth noting that the momentum matrix element ${\left|\hat{e}\;*\;p_{cv}\right|}^{2}$ is influenced by polarization, leading to variations in the gain coefficient *g* across different polarization states.

**Figure 5:**
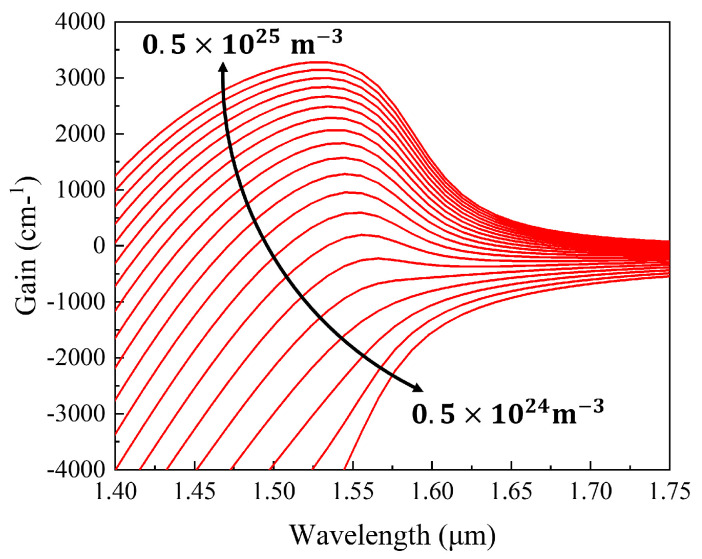
The material gain simulation curves.

We simulate the material gain curve of the epitaxial structure (see Figure 5), which has a carrier injection concentration ranging from 0.5 × 10^24^ m^−3^ to 0.5 × 10^25^ m^−3^. By comparing the gain curves at different injection concentrations, it is evident that the peak of the material gain curve is at 1550 nm, and under a high carrier injection, the gain curve further broadens. This indicates that the quantum well we designed can meet the requirements of a SOA for a broad gain spectrum range.

**Figure 6:**
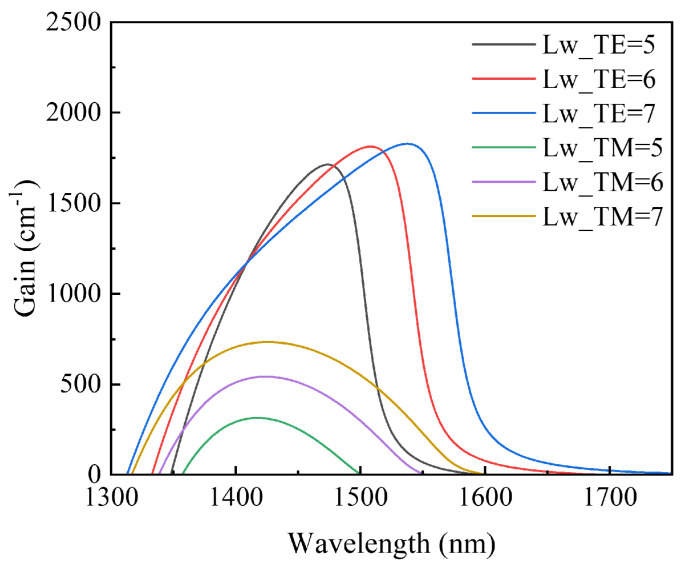
The material gain simulation curve of the TE mode and the TM mode at different quantum well widths.

We simulate the material gain of the TE mode and the TM mode at different quantum well widths (see Figure 6). From the simulation results, it is evident that with an increase in the quantum well width, the gain difference between the TE mode and the TM mode gradually decreases. This implies that wider quantum well structures can reduce the PDG of the device. However, considering the requirement to maintain a single-mode gain, as mentioned earlier, further widening of the quantum well was not adopted to reduce the PDG [10,11,12].

Another approach to reducing the PDG is by employing strained quantum well structures. By reintroducing strain effects in the material, the energy of the light-hole subband can be increased, thereby enhancing the mode gain of the TM mode. Although increasing the TM mode gain can effectively reduce the PDG of the SOA, it also affects the energy of the heavy-hole subband, leading to a decrease in mode gain and saturated output power of the TE mode. Hence, achieving a balance between high power and low PDG is challenging [13,14,15,16]. In this paper, some adjustments are made to the thickness and components of the quantum well to make a difference in the gain saturation for different modes without affecting the power in order to reduce the polarization sensitivity of the device, the details of which are further discussed in a later section.

### 2.3. The Design of the Active Region Mode Modulation

The optical field confinement factor Γ in the active region is an important parameter of the epitaxial structure. It is defined as the ratio of the power accounted for by the optical field within the active region to the full power, and can be expressed as follows [9]:

(7) Γ=12∫activeReE×H∗∗z^dx12∫allReE×H∗∗z^dx.

where E × H^*^ represents the complex slope-printing vector. The optical field confinement factor *Γ* not only exerts influence on the distribution of the optical field inside the waveguide, but also significantly impacts the saturated output power and small-signal gain of the amplifier. The TE modes are primarily constrained by the transverse electric field and are more dependent on the constraints in the high refractive index region, so the optical field constraint factor is usually larger than in TM modes. The gain in TE modes dominate in high-power devices, so the main focus is on the mode field variation of TE modes in subsequent simulations.

**Figure 7:**
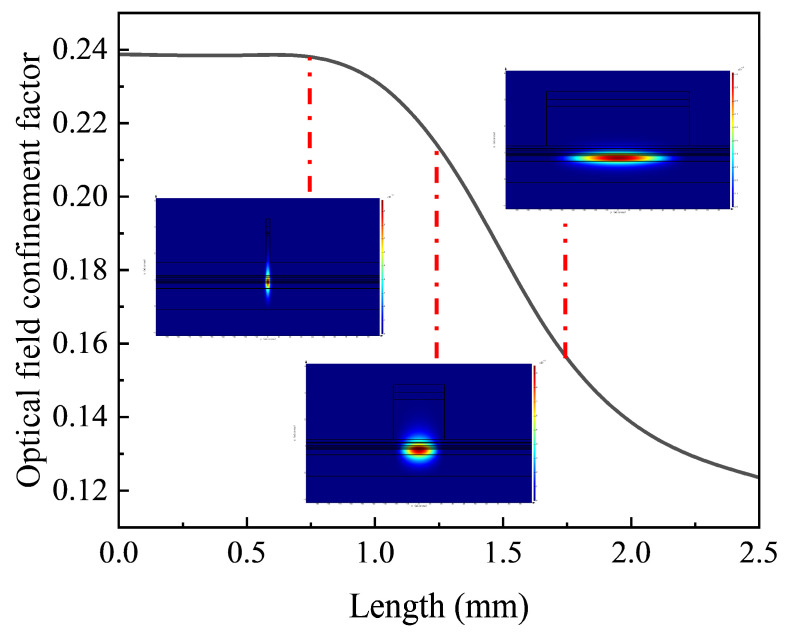
A simulation diagram of the optical field distribution changes in the device.

From the simulation of the optical field in the waveguide under different *Γ*s (see Figure 7), we can see that when the *Γ* is 0.2315, the optical field energy is mostly confined within the quantum well layer, indicating a structure that can achieve high small-signal gain. When the *Γ* is 0.168, it can be observed that the transmitted optical field leaks predominantly into the lower waveguide layer, expanding the optical field cross-sectional area. This would accommodate more energy, implying a potential for higher saturated output power. In the second-stage tapered waveguide region of the device, the low optical field confinement factor weakly constrains the optical field, allowing the optical field to expand into the lower waveguide layer. This greatly expands the cross-sectional area of the optical field to form a transmission optical field similar to that of a plate-coupled waveguide. This effectively increases the saturated output power of the device. 

Compared with the ridge waveguide, the gradually widening waveguide morphology of the tapered waveguide can expand the gain region in disguise [17,18,19,20]. Ideally, when the input light propagates along the tapered optical waveguide, a larger mode field is formed as the cross-sectional area of the waveguide expands, achieving a high gain and high saturated output power. Additionally, the large waveguide area also greatly reduces the thermal resistance of the waveguide and minimizes the occurrence of catastrophic optical damage (COD).

### 2.4. The Preparation of the Device

**Figure 8:**
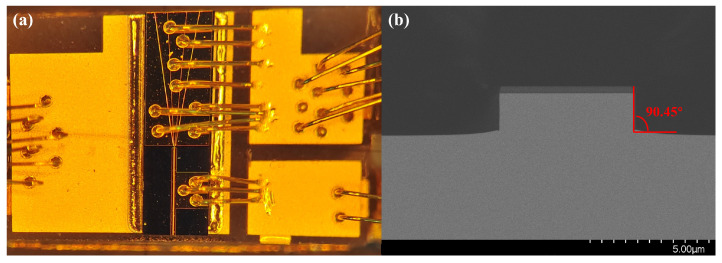
(**a**) Device package view; (**b**) SOA cross-section scanning electron microscope image.

The SOA structure to be tested is shown in Figure 8a. Distinct currents control two regions, and electrode isolation slots are incorporated between the ridged waveguide and tapered waveguide regions. The cross-section of the SOA along the BB’ in Figure 1 is depicted in Figure 8b, revealing a flat table etching of the waveguide with a lateral slope of 90.45°, as shown in the scanning electron microscope image.

Both cavity surfaces of the SOA are coated with an optical antireflection coating. This deliberate design not only augments the saturated output power of the SOA, but also diminishes noise, transmission loss, and the occurrence of COD. This comprehensive approach contributes to the overall enhancement of the device’s stability and performance.

## 3. Device Performance Testing and Analysis

### 3.1. The Configuration of the Test Platform

**Figure 9:**
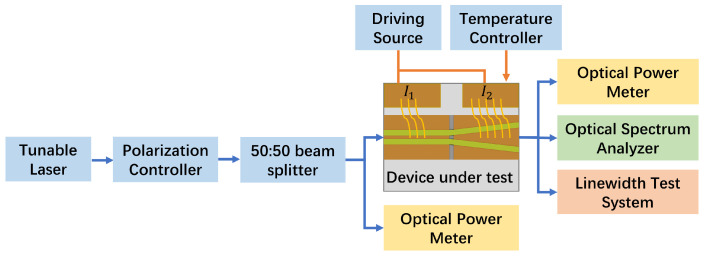
The testing platform.

To maintain precise temperature control throughout the testing process, the heat sink is attached to the thermoelectric cooler (TEC). The device test platform incorporates separate drive currents for the ridge waveguide and the tapered waveguide (see Figure 9). Two distinct currents are applied to control each. The light from the tunable laser is coupled with the ridge waveguide through a tapered fiber after passing through a polarization controller. During the test, we calculated the coupling loss of the tapered fiber and the chip into the core gain as part of the loss, and the input optical power was accurately measured by means of a 50:50 beam splitter at the input. Subsequently, the light underwent lens coupling at the opposite end, reaching the optical power meter, optical spectrum analyzer, and linewidth test system to evaluate device characteristics, including output power, gain, amplified spectrum, and linewidth spread. In our experiment, we used a tunable laser light source, EXFO T500S SCL tunable laser (EXFO, Quebec City, QC, Canada); an optical spectrum analyzer, Yokogawa AQ6370D (Yokogawa, Tokyo, Japan); and an optical power meter, Thorlabs’ PM400 (Thorlabs, Newton, NJ, USA).

Recognizing that the primary gain region of the device is situated in the second-stage conical waveguide area, the drive current (*I*_1_) for the ridge waveguide was held constant at 0.3 A throughout the testing process. The primary variable was the drive current (*I*_2_) for the conical waveguide, enabling an evaluation of the device’s amplification performance.

### 3.2. Testing for the Spectra of Amplified Spontaneous Emission

**Figure 10:**
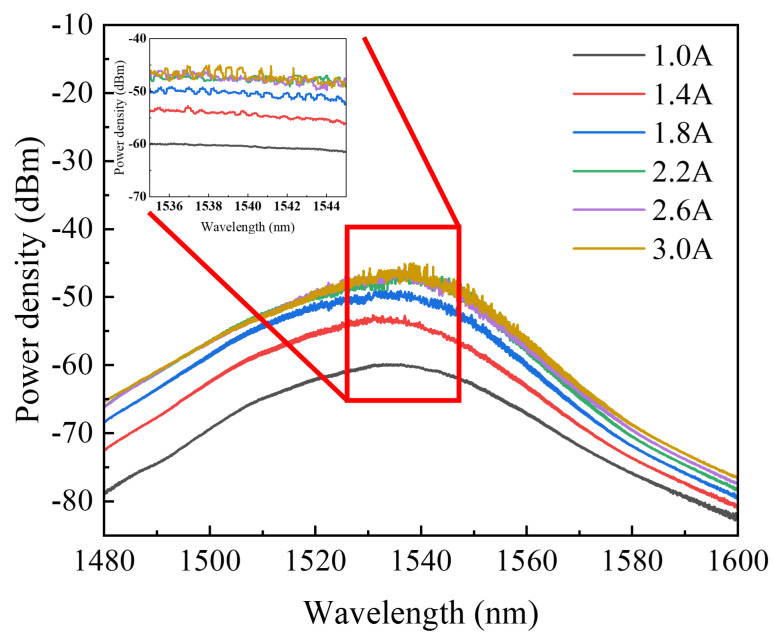
The amplified spontaneous emission spectra of the device under testing at different driving currents (*I*_2_).

In the experiment, the TEC temperature was controlled at 20 °C, and the amplified spontaneous emission spectrum (ASE) of the device under testing was measured using the spectrometer (see Figure 10). Subsequently, the *I*_2_ was adjusted to 1.0 A, 1.4 A, 1.8 A, 2.2 A, 2.6 A, and 3.0 A, respectively, to observe the variation of the ASE performance of the device. The ASE spectra provide insights into the amplification performance of the SOA in the absence of input light. According to the traveling wave amplifier, ideal conditions dictate a reflectivity of 0 for the SOA cavity surface. However, due to an actual process mistake, some reflectivity was inevitably present at the end surface of the SOA, leading to Fabry–Pérot (F-P) oscillations manifested as gain perturbations on the ASE spectra. As the driving current increased, the perturbation of the F–P resonance, induced by the residual reflectivity at the end facet, gradually intensified. The ripple increased from 0.094 dB at 1.0 A to 1.4 dB at 1.4 A. As the gain approached saturation, the perturbation further amplified, resulting in a ripple exceeding 2 dB.

To mitigate the impact of perturbations caused by the F–P resonance on SOA performance, enhancing the transmittance of the permeation-enhancing film or optimizing the waveguide structure—such as employing a slant-tapered waveguide—can be considered. These measures aimed to improve waveguide transmittance and minimize the observed perturbations.

### 3.3. Testing for Single Wavelength Output Power and Gain

**Figure 11:**
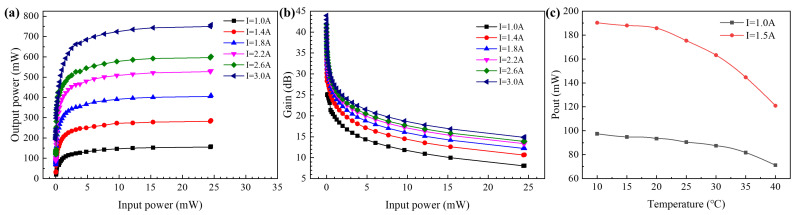
At the 1550 nm band: (**a**) the output power versus input power curve of the device under testing at different driving currents (*I*_2_); (**b**) the gain versus input power curve of the device under testing at different driving currents (*I*_2_); (**c**) the output power versus temperature curves of the device under testing at different drive currents (*I*_2_).

In the experiment, the wavelength of the seed source laser was set to 1550 nm and the TEC temperature was controlled at 20 °C. The *I*_2_ varied between 1.0 A, 1.4 A, 1.8 A, 2.2 A, 2.6 A, and 3.0 A to test the output power and gain performance of the amplifier (see Figure 11a,b). The experimental results show that the output power grows rapidly with an increase in input power at *I*_2_ of 3.0 A, but gradually approaches saturation when the input power exceeds 4 mW. As *I*_2_ increased, the output power of the device to be tested also increased significantly, and finally, the output power of the device to be tested was 757 mW when the input power was 25 mW. It can be seen that the second tapered waveguide section effectively increased the output power of the device. The relationship curve between gain and input power showed that the gain of the device to be tested was 27.32 dB when the *I*_2_ was 3.0 A and the input power was 0 dBm, but the gain gradually decreased with an increase in the input power, and dropped to 18.75 dB when the input power was 10 dBm. The temperature stability of the device was tested by adjusting the TEC temperature at different drive currents (see Figure 11c). At 10 °C, 20 °C, 30 °C, and 40° C, the output powers are 190.32 mW, 185.78 mW, 163.24 mW, and 120.94 mW, respectively. From the figure, it can be seen that the power of the device is more stable at a room temperature below 20 degrees Celsius and has a significant attenuation as the temperature rises. At a drive current of 1.5 A, the power of the device at 40 degrees Celsius is reduced to about 63% of the room’s temperature. The performance stability of the device was poor at high temperatures, for which further adjustments to the quantum well components were needed to improve the high-temperature operating characteristics of the device.

### 3.4. Testing for the Amplified Spectrum and Gain Curve

**Figure 12:**
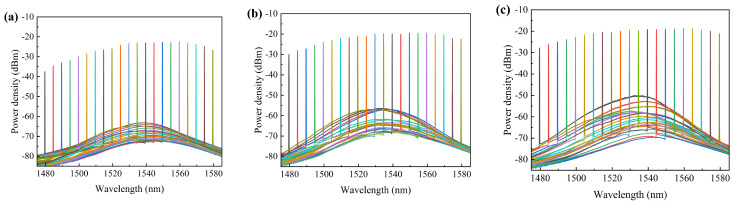
**The** amplified spectra of the device at (**a**) *I*_2_ = 1.0 A, (**b**) *I*_2_ = 1.5 A, and (**c**) *I*_2_ = 2.0 A.

In the experiment, the *I*_2_ varied between 1.0 A, 1.5 A and 2.0 A, respectively. The TEC temperature was controlled at 20 °C, and the output power of the seed source laser was set to 6 dBm. The accuracy of the Yokogawa AQ6370D optical spectrum analyzer was set to HIGH2 mode, 0.1 nm. The wavelength of the seed source laser was tuned, and the tuned amplified spectra of the amplifier were tested (see Figure 12).

The experimental results demonstrate that the device under testing exhibits a significant gain in the 1480–1580 nm band range. Throughout the tuning band, the side-mode rejection ratio of the device under testing remained consistently higher than 50 dB. Upon comparing the ASE spectra above, it was evident that the background noise was effectively suppressed when the SOA was powered by the seed source laser. However, at higher input power levels, the background noise was not further suppressed, due to SOA saturation. To further mitigate the background noise in the spectra, techniques such as adjusting the SOA temperature can be employed [21].

**Figure 13:**
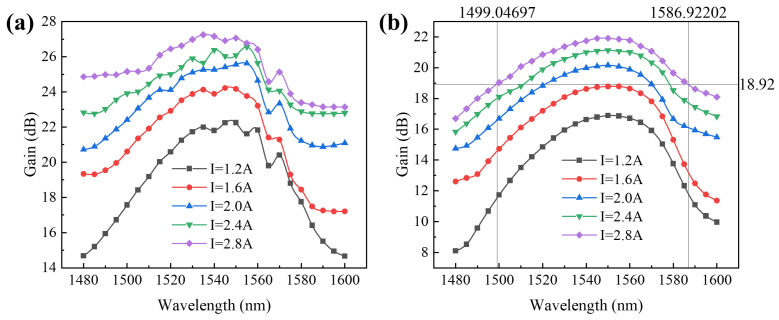
The gain curves of the device under testing between 1480 and 1600 nm with different driving currents (*I*_2_). (**a**) Seed source laser power Pin = 0 dBm; (**b**) seed source laser power Pin = 6 dBm.

In the experiment, the TEC temperature was maintained at 20 °C. The power of the seed source laser was set to 0 dBm and 6 dBm, while the *I*_2_ was varied at 1.2 A, 1.6 A, 2.0 A, 2.4 A, and 2.8 A. Subsequently, we tuned the wavelength of the seed source laser to investigate the amplifier’s gain bandwidth (see Figure 13). 

The experimental findings reveal that the device under testing operated in an unsaturated state at an input power of 0 dBm. The device attained a maximum gain of 27.25 dB when the *I*_2_ was set to 2.8 A. When the input power was increased to 6 dBm, the gain curve became smoother, and the maximum gain was 21.92 dB, with a 3 dB gain bandwidth of 88 nm observed when the *I*_2_ was 2.8 A. Although the gain in the near-saturated state was reduced compared to the unsaturated state, the gain bandwidth was further broadened. Optimization of the drive current and temperature control can enhance this bandwidth.

### 3.5. Testing for Line-Width Broadening Characteristics

**Figure 14:**
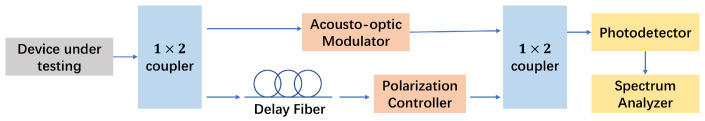
Structural schematic of delayed self-heterodyne linewidth test system.

This paper utilizes a delayed self-heterodyne linewidth test system to assess the linewidth broadening of the device under testing (see Figure 14). Following Lorenz fitting on the beat frequency peak, half of the 3 dB bandwidth of the Lorenz curve represents the linewidth of the device under testing.

**Figure 15:**
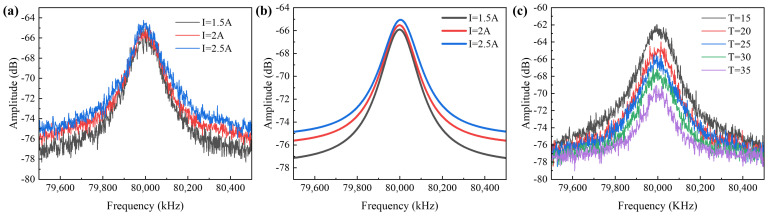
The beat frequencies of Lorentz fitting curves for linewidth at (**a**) different currents. (**b**) Rafter-fitting the data in (**a**). The resulting linewidths are 77.8 kHz, 78.65 kHz, and 80.25 kHz at *I*_2_ = 1.5 A, 2.0 A, and 2.5 A. (**c**) The linewidths at different temperatures are 77.8 kHz, 78.65 kHz, and 80.25 kHz at 15 °C, 20 °C, 25 °C, 30 °C, and 35 °C.

In the experiment, the power of the seed source laser was set to 6 dBm and the TEC temperature-controlled at 20 °C. The *I*_2_ varied between 1.5 A, 2.0 A, and 2.5 A, respectively, and the linewidth of the SOA output was measured (see Figure 15) using a delayed self-heterodyne linewidth test system. Although a high level of noise is present in the spectrometer waveform, a more accurate linewidth result can be obtained by Lorentz-fitting the curve with Origin. The Lorentzian fitting results revealed linewidths of 77.8 kHz, 78.65 kHz, and 80.25 kHz for currents of 1.5 A, 2.0 A, and 2.5 A, respectively. Since the input signal linewidth of the SOA was tested at 75.45 kHz, it can be observed that the amplified signal linewidths widened by factors of 1.031, 1.042, and 1.064, respectively, indicating that the linewidth broadened with an increase in current.

The variation of SOA linewidth at different temperatures showed output linewidths of 78.35 kHz, 78.95 kHz, 80.12 kHz, 82.65 kHz, and 83.95 kHz at 15 °C, 20 °C, 25 °C, 30 °C, and 35 °C, respectively (see Figure 15). The data suggests that the SOA output linewidth broadens as the temperature rises, consistent with the theoretical derivation mentioned earlier. Notably, the linewidth at 2.5 A is only 1.031 times the linewidth at 1.5 A, which is much smaller than the reported linewidth broadening of about 2 times reported by Shawki, Heba A’s research group [22].

### 3.6. Testing for Polarization Characteristics

**Figure 16:**
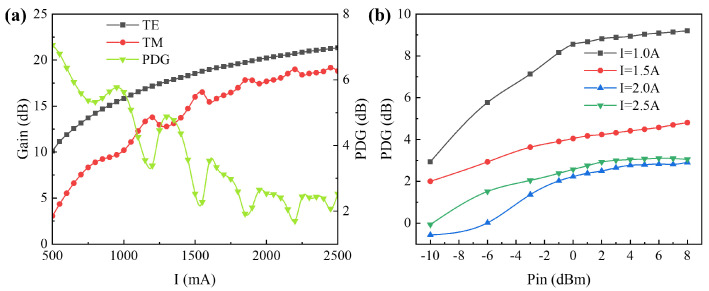
(**a**) The curve of the TE and TM mode gain of the device and the PDG at different driving currents (*I*_2_). (**b**) The relationship curve between the PDG of the device under testing and the seed source power.

In the experiment, the seed source laser power was set to 0 dBm, and the wavelength was set to 1550 nm. The different polarization states of the seed source laser input were controlled by manipulating the three-ring polarization control. By selecting the polarization state with the maximum and minimum gains, we obtained the curve of the TE and TM mode gain of the device and the PDG at different *I*_2_s (see Figure 16).

The experiments revealed that at low driving currents when the output power was not saturated, a substantial gap existed between the TE mode gain and the TM mode gain of the device, resulting in a PDG of 7.058 dB. As the driving current increased, the TE mode approached saturation first, leading to a slowdown in TE mode gain growth, and consequently, a gradual decrease in PDG. When the *I*_2_ was 2.5 A, the PDG was reduced to approximately 1.8 dB, indicating a low polarization sensitivity under saturated operating conditions. 

The overall PDG trend under different driving currents increased with the power of the seed source laser (see Figure 16b). Beyond a seed source laser power of 0 dBm, both the TE mode and the TM mode tend to saturate, and the PDG stabilizes. At a low driving current, where the gains of the TE mode and the TM mode have not reached saturation, the gap widens rapidly, leading to a rapid increase in PDG. However, when the driving current exceeds 2.0 A, the TE mode tends to saturate. Despite additional increments in the seed source laser power, the PDG stabilized at approximately 2.8 dB.

**Figure 17:**
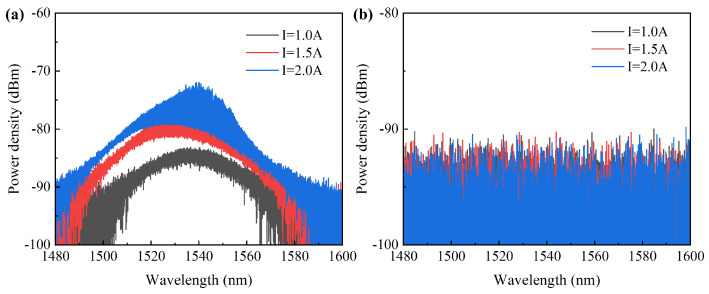
(**a**) TE mode ASE; (**b**) TM mode ASE.

In the experiment, the wavelength of the seed source laser was 1550 nm with a power of 6 dBm. *I*_2_ varied between 1.0 A, 1.5 A, and 2.0 A. The different polarization states of the ASE of the SOA exhibit a high polarization–extinction ratio (see Figure 17). The components are almost all TE modes, and there is almost background noise only in the TM modes. The polarization–extinction ratio of the ASE is 13 dB at a driving current *I*_2_ of 2 A.

**Figure 18:**
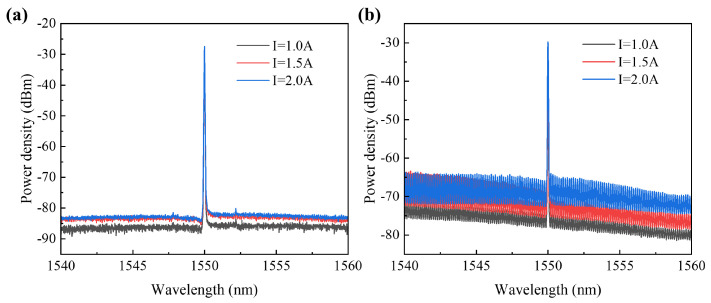
(**a**) TE mode amplified spectrum; (**b**) TM mode amplified spectrum.

The amplifier output spectra of the TE mode and TM mode showed that in the TE mode spectrum, the wavelength of the seed source laser completely dominated (see Figure 18). Compared to the ASE above, the amplified spectrum of the TE mode effectively suppressed the background noise, with the spectral side–mode suppression ratio exceeding 53 dB at different currents.

It was observed that in the spectrum of the TE mode, the wavelength of the seed laser completely dominated, and the noise was effectively suppressed, with a sidelobe suppression ratio exceeding 53 dB at different currents. In contrast, in the spectrum of the TM mode, the wavelength of the seed laser did not fully dominate, resulting in significantly higher noise compared to the TE mode spectrum, with a sidelobe suppression ratio of only around 40 dB. This indicates that the gain of the device for the TM mode is noticeably lower than that for the TE mode in an unsaturated state. Additionally, the noise waveform exhibited resonant characteristics, suggesting that insufficient transmittance from the device’s end facet for the TM mode may have caused some of the TM mode light to resonate within the amplifier cavity, resulting in relatively strong noise.

## 4. Discussion

The performance parameters of the SOA designed for this study are compared with those of previously reported SOAs in the literature, focusing, in particular, on the modulation section and the tapered section of the three-stage master oscillator power amplifier. The comparison results are summarized in Table 2 [23,24,25,26,27,28,29,30,31]. Comparatively, the SOA designed for this study demonstrates notable advantages in both output power and gain. Under a driving current of *I*_1_ = 0.3 A and *I*_2_ = 3.0 A, the SOA achieves an output power of 757 mW. The device achieves a balance between high gain and high output power, with a gain bandwidth greater than that of current commercial devices on the market. The device showed a stable performance at about 100 h of operation, however, longer aging tests need to be conducted in subsequent experiments.

The device under testing exhibits noticeable noise transmission in the TM mode, possibly due to two reasons. First, the gain of the TM mode is still significantly lower than expected, failing to completely suppress the background noise of the SOA. Second, the higher reflectivity of the device’s end facet to the TM mode may lead to resonant formation in some TM modes, resulting in increased noise levels. To further reduce noise and enhance the SOA’s output power, adjustments will be made from both the end facet and waveguide structure perspective. Fine-tuning the anti-reflective coating design on the end facet can further improve the transmittance of the TM mode, aiming to boost the output power and reduce the PDG. Additionally, refining the tapered waveguide structure and adopting a 7° tilted taper design can further enhance the device’s transmittance, which is beneficial for increasing the output power and reducing transmission losses. However, the impact of the tilted taper on the polarization needs further verification through simulation and experimentation.

## 5. Conclusions

This paper presents the design and fabrication of a 1550 nm high-power, low-linewidth broadening SOA based on active region mode control, with a detailed characterization of its performance parameters. The experimental results demonstrate that under a drive current of *I*_1_ = 0.3 A, *I*_2_ = 3.0 A, and a temperature of 20 °C, the SOA exhibits a 3 dB gain bandwidth of 88 nm. At a wavelength of 1550 nm and an ambient temperature of 20 °C, the output power reaches 757 mW when the input power is 25 mW, and the gain is 21.92 dB when the input power is 4 mW. With the laser power at 0 dBm, the output power is 540 mW, achieving a gain of 27.32 dB. The linewidth broadening is only 1.064 times, and the polarization-dependent gain is less than 3 dB near saturation, indicating low polarization sensitivity. Future work will focus on further optimizing the noise, saturated output power, and polarization-dependent gain of the device to meet the demand for high-power, high-gain, wide-gain spectrum, and narrow-linewidth SOAs for LiDAR applications, especially in frequency-modulated continuous-wave LiDAR.

## Figures and Tables

**Table 1:** A detailed description of the SOA’s composition.

Layer	Material	Repeat	Thickness (nm)	Doping Concentration
16	GaInAs	/	200	p-Zinc
15	GaInAsP	/	50	p-Zinc
14	InP	/	100	p-Zinc
13	InP	/	1500	p-Zinc
12	GaInAsP	/	20	p-Zinc
11	InP	/	50	p-Zinc
10	AlGaInAs	/	60	p-Zinc
9	AlGaInAs	/	60	Undoped
8	AlGaInAs	/	10	Undoped
7	AlGaInAs	5	6	Undoped
6	AlGaInAs	5	10	Undoped
5	AlGaInAs	/	60	Undoped
4	AlGaInAs	/	60	n-Silicon
3	AlGaInAs	/	10	n-Silicon
2	InP	/	500	n-Silicon
1	InP	/	300	n-Silicon

**Table 2:** A comparison of the SOA performance parameters.

References	Research Unit	I_1_/I_2_ (A)	Output Power (mW)	Gain (dB)	Linewidth Expansion
Ref. [23]	Rose Hulman Inst Technol	0.3/4.0	400	~16.4	No test
Ref. [24]	III V Lab	0.3/3.0	380	~22.8	No test
Ref. [25]	Univ Politecn Madrid	0.5/3.5	300	~15.0	No test
Ref. [26]	Univ Politecn Madrid	0.5/3.5	250	No test	No test
Ref. [27]	Rose Hulman Inst Technol	0.3/3.0	375	~15.7	No test
Ref. [28]	Univ Politecn Madrid	0.3/4.0	380	No test	No test
Ref. [29]	III V Lab	0.3/3.0	630	No test	No test
Ref. [30]	Univ Glasgow	0.3/0.8	210	~9.6	No test
Earlier work [31]	CIOMP	0.3/2.5	419	26.23	1.15
This paper	CIOMP	0.3/3.0	757	27.32	1.031

## Data Availability

Data are contained within the article.
